# Wound Healing Activity of Topical Application Forms Based on Ayurveda

**DOI:** 10.1093/ecam/nep015

**Published:** 2011-05-26

**Authors:** Hema Sharma Datta, Shankar Kumar Mitra, Bhushan Patwardhan

**Affiliations:** ^1^Interdisciplinary School of Health Sciences, University of Pune, Ganeshkhind, Pune 411007, India; ^2^The Himalaya Drug Company, Makali, Bangalore 562 123, India; ^3^Manipal Education, 14 Airport Road, Manipal Towers, Bangalore 560 008, India

## Abstract

The traditional Indian medicine—Ayurveda, describes various herbs, fats, oils and minerals with anti-aging as well as wound healing properties. With aging, numerous changes occur in skin, including decrease in tissue cell regeneration, decrease in collagen content, loss of skin elasticity and mechanical strength. We prepared five topical anti-aging formulations using cow ghee, flax seed oil, *Phyllanthus emblica* fruits, *Shorea robusta* resin, *Yashada bhasma* as study materials. For preliminary efficacy evaluation of the anti-aging activity we chose excision and incision wound healing animal models and studied the parameters including wound contraction, collagen content and skin breaking strength which in turn is indicative of the tissue cell regeneration capacity, collagenation capacity and mechanical strength of skin. The group treated with the formulations containing *Yashada bhasma* along with *Shorea robusta* resin and flax seed oil showed significantly better wound contraction (*P* < .01), higher collagen content (*P* < .05) and better skin breaking strength (*P* < .01) as compared to control group; thus proposing them to be effective prospective anti-aging formulations.

## 1. Introduction

Aging is a universal process that began with the origination of life many years ago. Skin being the largest organ of the body deserves special attention while considering the aging process. Skin aging is due to the conjunction of intrinsic factors (chronological aging) and extrinsic factors (fundamentally photo aging). In elderly people, cells are less likely to proliferate, they have shorter life spans, are less responsive to cytokines and epithelialization of epithelial skin tissue is longer. Proteases associated with intrinsic aging (metalloproteases) appear increased and less challenged which may predispose to tissue breakdown in the elderly people [1–3]. Thus, to assess integrity of the skin structure, collagenation (collagen synthesis), epithelialization, connective tissue (matrix) deposition and tissue cell regeneration, wound healing animal models can be used [4, 5]. Several studies have been reported to evaluate wound healing activity using different models [6–8]. We have studied the effect of selected topical application forms on tissue regeneration ability and functional status of skin in wound healing models by measuring wound contraction, collagen estimation and breaking strength of the skin.

Ayurveda remains one of the most ancient and yet alive tradition practiced widely in India, Sri Lanka and other countries that have a sound philosophical and experiential basis [9]. *Atharvaveda* (around 1200 BC), *Charak Samhita* [10] and *Sushrut Samhita* (1000–500 BC) are main classics that give detailed descriptions of over 700 herbs. A scholarly description of the legacy of *Charaka* and *Sushruta* in contemporary idiom, best attempted with a commentary from modern medicine and science viewpoint, gives some glimpses of ancient wisdom [11]. Ayurveda and traditional Chinese medical system share many common approaches and have a long history of practice [12]. Ayurveda has several formulations for management of aging and related conditions. Ayurvedic literature describes more than 200 herbs, minerals and fats for skin care. We selected cow ghee (butter fat), flax seed oil, *Amalaki* fruit (*Phyllanthus emblica*), *Shorea robusta* resin and *Yashada bhasma* (zinc complex) for this study. Traditionally prepared cow ghee has two types of properties *Samshodhana* (cleansing or detoxifying) and *Samshana* (palliative). Such ghee alone or in combination with honey [13] is considered to be extremely useful for treating wounds, inflammatory swellings and blisters for promotion of quick healing. Ghee is used in many Ayurvedic traditional preparations and also as an ointment base. Flax seed oil is believed to bring mental and physical endurance by fighting fatigue and controlling aging process. It has properties like *Madhura* (balances the skin pH), *Picchaila* (lubricous), *Balya* (improves tensile strength or elasticity of the skin), *Grahi* (improves moisture holding capacity of skin), *Tvagdoshahrit* (removes skin blemishes), *Vranahrit* (wound healing) and useful in *Vata* disorders including dryness, undernourishment, lack of luster/glow [14]. Both cow ghee and flax seed oil are rich sources of essential fatty acids (EFAs), which regulate prostaglandin synthesis and hence induce wound healing. Deficiency of EFA results in phrynoderma or toad skin, horny eruptions on the limbs, poor wound healing, etc. Omega-3 and Omega-6 EFAs are thus important for the maintenance of normal epidermal structure [15–18]. *Phyllanthus emblica* or *Amalaki* fruit is a rich source of vitamin C, which is a potent antioxidant [19]. It is foremost amongst the anti-aging drugs (*vayasthaprana*) or best amongst the rejuvenating herbs; it has properties like *Rasayana* (adaptogenic), *ajara* (usefulness in pre-mature aging), *ayushprada* (prolongs cell life), *sandhana karaka* (improves cell migration and cell binding) and *Kantikara* (improves complexion). Juice of the fresh fruit and ghee mixed together is a good restorative tonic. *Amalaki* helps in fighting many obstinate skin diseases [20]. *Shorea robusta* resin with Beeswax is used as a base for ointment of herbal extracts used in the healing of foot cracks, psoriasis and other chronic skin diseases. It is useful in wounds, ulcers, neuralgia, burns, pruritus and various skin problems. A combination of cow ghee and *Shorea robusta* resin is applied in rectal prolapse, haemorrhoids to control burning sensation and excess secretion. An external application made of herbal powders and oleoresin with cow ghee is advocated in blisters to control pain and swelling. Due to excellent healing property, a combination of selected herbal ingredients along with cow ghee and oleoresin is applied on burns and other injuries. It also has anti-inflammatory, antiseptic and anti-microbial properties useful in treatment of skin diseases both infectious and metabolic [21]. *Yashada bhasma* is a traditional herbomineral preparation mainly comprising of zinc which plays an important role in the normal functioning of skin as it influences the synthesis of collagen. Zinc would benefit the system undergoing rapid cellular division such as wound healing. Studies indicate the epithelization of burns may be improved by zinc treatment. Zinc markedly increases the stability of bio-membranes in general. Ayurvedic literatures describe the activities of *Yashada bhasma* as *Krimighna* (antimicrobial), *Kantikara* (improves complexion), *Rasayana* (rejuvenator) and *Grahi* (improves moisture holding capacity of skin). It also improves the binding power of the cells of skin soft tissues, improves cell migration and cell regeneration and hastens wound healing [22–24].

## 2. Materials and Methods

### 2.1. Test Material

Cow ghee was obtained from The Nilgiri Dairy Farm Ltd, Bangalore, India; flax seed oil was obtained from Arpitha Aromatics, Bangalore, India; *Amalaki* fruit extract (spray-dried juice) was obtained from Venkatesh Food Industries, Chhindwara, India; *Shorea robusta* resin was obtained from M/S Aditya Solvent and Chemicals, Bangalore, India; *Yashada bhasma* was obtained from M/S Uma Ayurvedics Pvt. Ltd, Kasganj, India. All the samples were identified and confirmed by the Pharmacognosy Department of the Research and Development Center of The Himalaya Drug Company, Bangalore, India. Further identification and authentication of test materials was done by routine or pharmacopoeial methods [25–30] based on parameters such as solubility, pH, loss on drying, ash content, refractive index, specific gravity, saponification value, acid value, iodine value, peroxide value, moisture content, heavy metal analysis, microbial load, zinc content and vitamin C content.

### 2.2. Chemicals

Alpha linolenic acid (ALA) reference standard was purchased from Sigma-Aldrich Inc., USA. All other chemicals and reagents used were analytical grade, unless otherwise specified.

### 2.3. Formulation of Topical Application Forms

We prepared five variants of the anti-aging topical application form comprising of the study materials in water and in oil emulsion cream. All the variants comprised of the base formulation consisting of *Shorea robusta* resin and flax seed oil in 1 : 4 proportion. The formula details of the cream variants are given in Table 1. Pharmaceutical grade de-mineralized water and permitted preservative system (methyl paraben+propyl paraben) were used in all test preparations. The stability testing conditions and parameters for the creams were generally based on ICH stability guidelines, WHO stability guidelines and Bureau of Indian Standards. The studies were done in two parts: quick assessment stability studies (from 48 h to 1 month) and accelerated stability studies (6 months). The quick assessment stability studies comprised of thermal stability testing (48 h at 45 ± 1°C/70% RH), freeze thaw testing (five cycles at –5 ± 1°C to 30 ± 1°C) and low temperature testing (1 month at 5°C). The physical parameters including phase separation and color change were observed. The samples were then subjected to accelerated stability conditions (40°C/75% RH) for 6 months and the physicochemical parameters included: pH, viscosity, % total fatty matter content, % water content and % residue. Microbiological stability studies included determination of TVC (total viable count), TFC (total fungal count) and PET (preservative efficacy test or challenge test) [31–34]. Percentage of ALA fatty acid content was also determined in accelerated stability studies.

### 2.4. Determination of ALA Content by Gas Chromatography

The ALA content of flax seed oil, cow ghee, *Shorea robusta* resin extract in flax seed oil and the anti-aging topical application forms was estimated using gas chromatography pharmacopoeial method [26]. The Shimadzu Gas Chromatograph, GC-14 B comprising of flame ionization detector (FID), GC Column—BP × 70 × capillary (SGE) and with s/w—CLASS-GC 10 (version 2.0) was used.

### 2.5. Chromatographic Conditions

Temperature conditions—Oven temperature—100°C, Injector temperature—130°C, Detector temperature—240°C. Initial 100–160°C at 8°C for 2 min, initial 160–190°C at 10°C for 3 min, initial 190–230°C at 18°C for 6.2 min, total run time—25 min. Gas flow conditions—carrier nitrogen—1 ml/min. Hydrogen—30 ml/min, oxygen—30 ml/min, split ratio—1 : 9.

### 2.6. Standard and Sample Preparation

Reference standard (ALA) 25–100 mg was weighed accurately in 125 mL round-bottomed flask. To this was added 10 mL of pure methanol (HPLC grade) and two to three drops of conc. H_2_SO_4_, and the solution refluxed for 3 h at 70°C. After refluxing the contents were cooled to room temperature. It was then extracted with 25 mL of petroleum ether twice. The petroleum ether extracts were collected, combined and filtered through Whatman filter paper (Grade-1, Cat. No 1001917) using anhydrous sodium sulphate. The petroleum ether was evaporated completely at 60°C and the residue dissolved in 5 mL of petroleum ether and made up to 10 mL in volumetric flask and injected into GC. For sample preparation, 2.0–2.5 g of the test sample was weighed accurately, and to this was added 20 mL of 0.5 N methanolic NaOH in a round-bottomed flask and refluxed for 30 min at 70°C using cooling condenser. After refluxing the contents were cooled to room temperature and to it was added 20 mL of water, 1.0 mL of conc. HCl and shaken well. The contents were transferred into separating funnel of capacity 250 mL and extracted with 50 mL of petroleum ether thrice. The petroleum ether extracts were collected, combined and filtered through Whatman filter paper using anhydrous sodium sulphate. The petroleum ether was evaporated completely at 60°C. The residue was taken in round-bottomed flask and to this was added 25 mL of methanol (HPLC grade), four to five drops of sulphuric acid and refluxed for 3 h .Then the method as described for standard preparation was followed. One to two microliters of the sample were injected in GC and the respective components were calculated as:

(1) $$\begin{array}{cc} \text{Percentage}\;\text{of}\;\text{ALA}\;\text{Content}=\frac{\text{Sample}\;\text{area}}{\text{std}\;\text{area}} & \\ \times \frac{\text{std wt}}{\text{sample}\;\text{weight}}\times \text{Dilution}\times \text{Purity}\;\text{of}\;\text{std}. & \end{array}$$

### 2.7. Evaluation of Wound Healing Activity

For evaluation of wound healing activity of NATAF creams, excision and incision wound models were used [35, 36].

### 2.8. Animals

Laboratory-bred (Department of Pharmacology of Research and Development Centre of Himalaya Drug Co., Bangalore, India) rats of Swiss Wistar strain weighing 275–325 g were used for the study. The rats were housed in polypropylene cage and maintained in standard laboratory conditions of temperature (22 ± 2°C) and light–dark cycle of 12 : 12 h. The animals were fed with synthetic pellet diet from Tetragon Chemie Pvt. Ltd, Bangalore, India and water *ad libitum* during the experiment. The Institutional Animal Ethics Committee (Reg. No 26/1999/CPCSEA) permitted the study for wound healing.

### 2.9. Group Classification

Animals were randomized into six groups of six animals each. The animals of Group 01 were not treated with any cream (to serve as the control group), the animals of Groups 02, 03, 04, 05 and 06 were treated with topical applications of the following creams—NATAF-001, NATAF-002, NATAF-003, NATAF-004, NATAF-005, respectively once daily on the wound area. For both excision wound model and incision wound model the animal groups were classified and treated in the same manner.

### 2.10. Excision Wound Model

The animals were weighed individually, anaesthetized with pentobarbitone sodium (35 mg/kg, intraperitoneal). The rats were inflicted with excision wounds as described by Morton and Malone [37]. The skin of the dorsolateral flank area was shaved with an electrical clipper. After wound area preparation with 70% alcohol, the skin from the predetermined shaved area was excised to its full thickness to obtain a wound area of about 500 mm. Excision wounds were created on the dorsal thoracic region 1.5 cm from the vertebral column on either side. Hemostasis was achieved by blotting the wound with a cotton swab soaked in normal saline. The respective creams were topically applied on the wound area of the animals of respective groups once a day till complete epithelization; starting from the day of operation. Percentage wound contraction and collagen estimation parameters were studied.

### 2.11. Percentage Wound Contraction

Wound healing is a complex process that results in the contraction and closure of the wound and restoration of the functional barrier. Contractions, which contribute to wound closure, were studied on alternate days from Day 1 to Day 9, that is, starting from the day of operation till the day of complete epithelization by tracing the raw wound on a transparent sheet. The wound area was measured by retracing the wound by the UTHSCSA software Image Tool (Version 3.0.100.0). Falling of the eschar (dead tissue remnants) without any residual raw wound was considered as end point of complete epithelization. Percentage wound contraction was calculated as:

(2) $$\begin{array}{cc} & \text{Percentage}\;\text{wound}\;\text{contraction}\;\text{on}\\ & N\text{th}\;\text{day}=100-\frac{\text{Wound}\;\text{area}\;\text{on}\;N\text{th}\;\text{day}}{\text{Wound}\;\text{area}\;\text{on}\;1\text{st}\;\text{day}}\times 100. \end{array}$$

### 2.12. Collagen Estimation (Hydroxyproline Content)

Wound tissues were analyzed for hydroxyproline content, which is basic constituent of collagen. The collagen composed of amino acid (hydroxyproline) is the major component of extra-cellular tissue, which gives strength and support. Breakdown of collagen liberates free hydroxyproline and its peptides. Measurement of hydroxyproline hence can be used as a biochemical marker for tissue collagen and an index for collagen turnover [38]. For preparation of protein hydrolysate, 50 mg of tissue sample in 1.0 mL of 6.0 N HCl was weighed and sealed in screw-capped glass tube. The tubes were autoclaved at 15 1.056 kilograms per square centimetre for 3 h. The hydrolysate was neutralized to pH 7.0 and brought to the appropriate volume (filtered if necessary). Test tubes marked as sample, standard and blank were taken. One milliliter of test sample was added to test tubes marked as sample, 1.0 mL of DM water to test tubes marked as blank and 1.0 mL standard solutions to test tubes marked as standard. One milliliter of 0.01 M copper sulphate solution was added to all the test tubes followed by the addition of 1.0 mL of 2.5 N sodium hydroxide and 1.0 mL of 6% hydrogen peroxide. The solutions were occasionally mixed for 5 min and then kept for 5 min in a water bath at 80°C. Tubes were chilled in ice-cold water bath and 4.0 mL of 3.0 N sulphuric acid was added with agitation. Two milliliters of *p*-(dimethylamino)benzaldehyde was then added and heated in water bath at temperature 70°C for 15 min. The absorbance was measured at 540 nm using Synergy HT Multi-Detection Microplate Reader (MDMR). The concentration of the sample was calculated as:

(3) $$\begin{array}{cc} \text{Concentration}\;\text{of}\;\text{the}\;\text{sample} & =\frac{\text{OD}\;\text{of}\;\text{the}\;\text{sample}}{\text{OD}\;\text{of}\;\text{standard}}\\ & \times \text{Concentration}\;\text{of}\;\text{standard}. \end{array}$$

### 2.13. Incision Wound Model

The animals were weighed individually, anaesthetized with pentobarbitone sodium (35 mg/kg, intraperitoneal) and the skin of the dorsolateral flank area was shaved with an electrical clipper. After wound area preparation with 70% alcohol, two para vertebral long incisions were made through the skin at a distance of about 1.5 cm from the midline on either side. Each incision made was 5 cm long and the parted skin was stitched with interrupted sutures using black braided silk surgical suture (size 3X0) and a curved needle (No. 11) at 1.0 cm interval. The respective creams were topically applied on the wound area of the animals of respective groups once a day till 7th day starting from the day of operation. The sutures were removed on 7th day and the skin breaking strength of the healed wound was measured on 8th day.

### 2.14. Breaking Strength

One of the most crucial phases in dermal wound healing is the progressive increase in biomechanical strength of the tissue; the mechanical properties of the skin are mainly attributed to the function of the dermis in relation to the structure of collagen and elastic fiber networks. Breaking strength of the healed wound is measured as the minimum force required to break the incision apart. Skin breaking strength gives an indication of the tensile strength of wound tissues and represents the degree of wound healing [39].

Tensile strength has commonly been associated with the organization, content and physical properties of the collagen fibril network. Tensile strength is the resistance to breaking under tension; it indicates how much the repaired tissue resists breaking under tension and may indicate in part the quality of the repaired tissue. After removal of skin sutures on postoperative Day 7, gradually increasing weight was applied to one side of the wound while the other side was fixed. The weight that completely separated the wound from the incision line is considered to be the breaking strength. The sutures were removed on the 7th day after wounding and the breaking strength was measured on the 8th day. The mean breaking strength on the two para vertebral incisions on both sides of the animals were taken as the measures of the breaking strength of the wound of the individual animal.

### 2.15. Statistical Analysis

All the values were expressed as mean ± SEM. The values were analyzed using one-way analysis of variance (ANOVA) followed by Dunnett's *post hoc* multiple comparison test to establish statistical significance. The analysis was performed using Graph Pad Prism software (Version 4.0).

## 3. Results

The physicochemical analysis results for the study materials are tabulated in Table 2. The quick assessment stability studies results for the cream variants showed no phase separation or color change for any of the creams and hence were subjected to accelerated stability conditions (40°C/75% RH) for 6 months. The pH of all the variants was stable in the range of 5.0–6.5, which is nearer to the normal physiological pH to ensure better acceptability on application to the skin. Viscosity of all the variants showed a general trend in rise to about 200000–300000 cps over a period of 6 months which is acceptable for the non-Newtonian systems. Other parameters like TFM (total fatty matter), water content, residue, remained constant. Microbiologically, all the variants were stable with TVC and TFC count less than 10 (cfu/g) and all the batches passed the PET analysis which confirms the efficacy of the preservative system.

### 3.1. ALA Content

The GC retention time for ALA was found to be 17.531 min. The percentage ALA content for flax seed oil and cow ghee was found to be 6.42% and 1.23%, respectively, for *Shorea robusta* extract in flax seed oil it was found to be 5.5–6.0%. For the cream variants, the percentage ALA content remained stable for 6 months even in accelerated stability studies as depicted in Table 3.

### 3.2. Excision Wound Model

**Figure 1:**
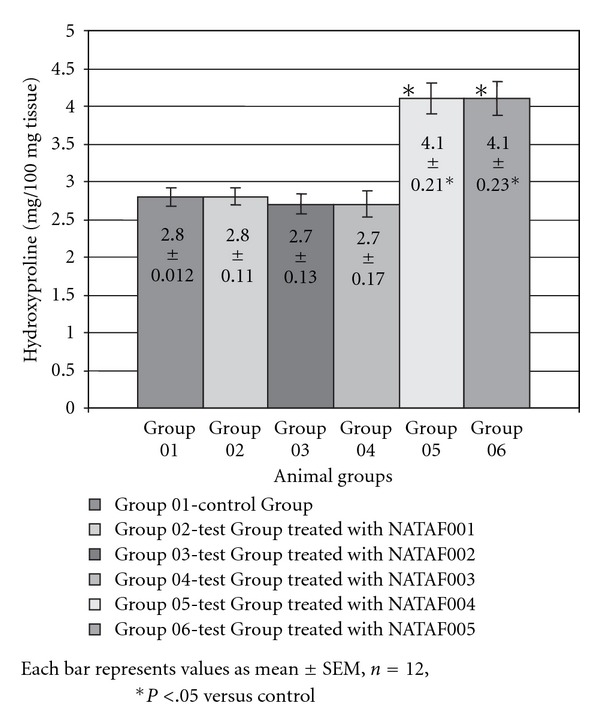
Hydroxyproline content of animal groups in excision wound model.

Wound contraction ability in excision model was measured at different time intervals till complete wound healing took place.  Table 4 depicts the effect of topical application of NATAF cream variants on percentage wound contraction in excision wound model. Group 05 (treated with NATAF 004) and Group 06 (treated with NATAF 005) exhibited significant (*P* < .01) increase in the percentage of wound contraction as compared to the untreated control on Day 7 and Day 9. After complete wound healing the hydroxyproline content which is indicative of the collagen turnover was determined in the treatment groups and control group. The results presented in Figure 1 clearly depicts that in treatment Group 05 (treated with NATAF 004) and Group 06 (treated with NATAF 005) the hydroxyproline content was significantly higher (*P* < .05) compared to the control Group 01.

### 3.3. Incision Wound Model

**Figure 2:**
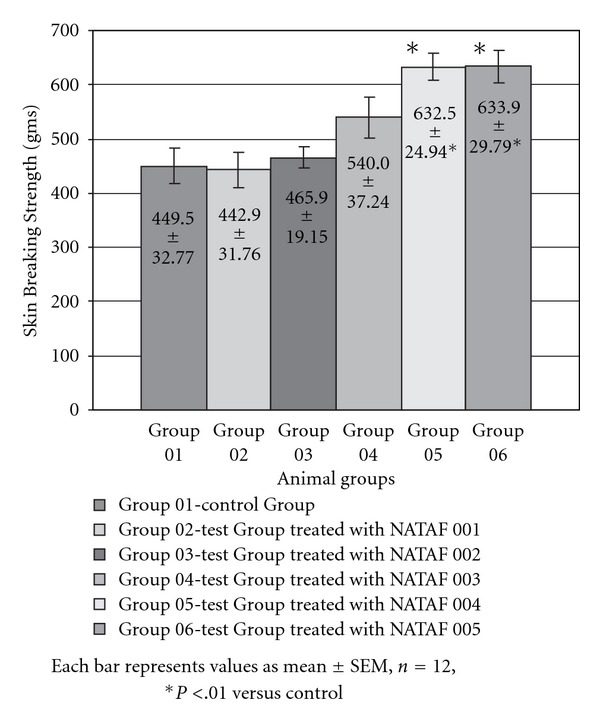
Skin breaking strength of animal groups in incision wound model.

The results of the measurement of skin breaking strength on 8th day post operation in incision wound healing model are depicted in Figure 2. The skin breaking strength in the animals of the treatment Groups 05 and 06 was significantly (*P* < .01) greater than that of the animals of the untreated group or control Group 01 thus showing enhanced collagen synthesis. For the animals of the other treated Groups 02, 03 and 04 there was not significant difference in the breaking strength when compared to the control Group 01. The breaking strength ultimately depicts the tensile strength, thus showing a significant increase in the tensile strength of the skin tissues in animals of Group 05 (treated with NATAF 004) and Group 06 (treated with NATAF 005), whereas the other groups showed skin tensile strength similar to that of the control group.

## 4. Discussion

Skin aging is a complex phenomenon and the most common amongst the visible signs of aging are wrinkles, pigmentation, dryness and laxity [40, 41]. As a consequence of aging, skin tissue cell regeneration capacity declines and the connective tissue (elastin, collagen, extra-cellular matrix) dwindles. The skin moisture levels, visco-elasticity and mechanical strength also decrease. Phenomena of aging provokes decline in defense, healing perception mechanisms and in thermo regulation of the skin tissue [42–45]. Most common external therapies to combat aging include use of anti-aging topical application forms, which are designed to delay and/or reverse signs of aging. In this study we formulated five variants of topical application forms with the study materials (flax seed oil, cow ghee, *Amalaki* fruit extract, *Shorea robusta* resin and *Yashada bhasma*) in a w/o emulsion cream form. The study materials were chosen based on the leads from Ayurvedic literature. For efficacy evaluation of the formulated anti-aging cream variants (NATAF creams), we used excision and incision wound healing models and studied suitable and relevant parameters such as wound contraction, hydroxyproline content (collagen content) and skin breaking strength which generally indicate the rate of tissue cell regeneration, amount of collagen or rate of collagenation and tensile strength of the skin.

**Figure 3:**
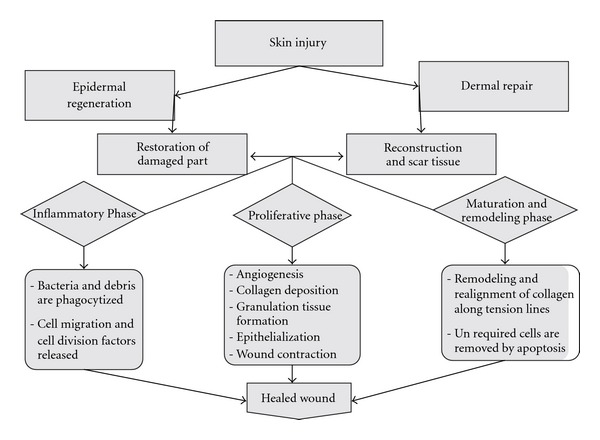
Mechanism of wound healing.

The normal response of an organism to injury or wound is either regeneration (the complete restoration of the damaged part) or repair (the reconstruction of the injured region). The scar tissue is appropriately covered, by an epithelium at the site of injury. When skin is injured or wounded the dermis responds primarily to repair while the epidermis responds to regeneration; the collective response of the skin to injury is termed as wound healing. Mechanisms involved in wound healing are epithelialization, contraction, connective tissue (matrix) deposition (Figure 3). Epithelialization is a process where keratinocytes migrate from the lower skin layers and divide. Contraction is the process where the wound contracts, narrowing or closing the wound. Connective tissue and matrix deposition is the process where fibroblasts come into the area and produce new matrix and collagen is laid down over and amongst this amorphous material. The epithelial tissues may then migrate over this. The matrix consists of collagen, elastin, fibronectin, laminin, hyaluronic acid, proteoglycans. These structures and chemicals give strength and support, allow expansion and contraction, provide a surface for cell movement, and help necessary chemical reactions to occur. Thus by measuring wound contraction, collagen estimation and breaking strength of the skin in wound healing models the integrity of the skin structure, collagen synthesis, epithelialization, connective tissue (matrix) deposition, tissue cell regeneration can be assessed [46–48].

Wound healing generally requires support at three levels. First, improving general resistance and support mechanisms that could be obtained from rejuvenative, adaptogenic, palliative, antioxidant, cleansing, detoxifying, buffering and lubricous activities. Second, stimulating the repair and regenerative mechanisms to prolong cell life, cell migration and cell binding, remove skin blemishes, and improve tensile strength or elasticity of the skin, improved moisture holding capacity of skin. Third, therapeutic and nutritional activities including anti-inflammatory, antiseptic and antimicrobial, protein and collagen synthesis and increased stability of bio-membranes. Antioxidants can interfere with the oxidation process by reacting with free radicals, chelating catalytic metals and also by acting as oxygen scavengers. Free radicals and other reactive oxygen species (ROS) are considered to be important causative factors in the aging process. Oxidative stress also plays an important role in impaired wound healing. Botanicals with anti-oxidant or free radical scavenging activity thus can play a significant role in healing of wounds [49].

We searched for such properties from Ayurvedic literature and practice following the reverse pharmacology path [50, 51]. This led us to a shortlist of materials from almost 200 different options. We used a blend of *Shorea robusta* resin extract in flax seed oil as a base or platform formulation. We prepared four additional variants by adding cow ghee, *Amalaki* fruit extract and *Yashada bhasma*. Although, there have been few reports indicating untoward effects of Ayurvedic bhasma preparations containing metals, we still chose to use *Yashada bhasma* as source of zinc. There are sufficient pharmacoepidemiological evidences [52] to show that traditionally prepared *bhasmas* are safe [53]. We have ensured authenticity and chemical consistency of all the materials used. Physicochemical studies on *Shorea robusta* resin, cow ghee, *Yashada bhasma* and flax seed oil were done following pharmacopoeial standards. Correct botanical identification of *Amalaki* was done by routine pharmacognostic tools as also by using molecular markers [54]. As given in the introductory part of this article, the additional ingredients have putative activities that are important for wound healing activity. We hypothesized that such a combination would have a synergistic activity and put all the five test materials through appropriate experimental tests.

The base formulation did not show statistically significant activity in any of the test models. Amongst other treatment groups, Groups 05 and 06, treated with NATAF 004 and NATAF 005, respectively, showed significantly greater wound contraction, higher collagen content and increased skin breaking strength as compared to the control group. All the other treatment groups, Groups 02, 03 and 04 treated with NATAF 001, NATAF 002 and NATAF 003, respectively, had no statistically significant augmenting effect on the process of wound healing. The positive effect of NATAF 004 and NATAF 005 creams can be attributed to presence of *Yashada bhasma* that has probably acted synergistically. *Amalaki* fruit extract and cow ghee addition showed marginal improvement but was not statistically significant. Thus, the common ingredient synergistically facilitating wound healing process appears to be *Yashada bhasma*. This is in agreement with its properties described in Ayurveda such as *Vranasamsravarodhanam* (improves cell migration, cell regeneration and hastens wound healing), *Slemshakalesankochakrit* (improves the binding power of the cells of skin soft tissues) and *Rasayana* (rejuvenator). *Yashada bhasma* needs suitable vehicle for application and therefore we used the base formulation. However, there may be an independent study needed to evaluate activity of *Yashada bhasma* per se. Flax seed oil and *Shorea robusta* resin have *Balya* (improves tensile strength/elasticity of the skin) and *Vranahrit* (wound healing), properties as described in Ayurveda. Furthermore, we did not observe any significant correlation found between ALA content and the wound healing activity of the anti-aging cream variants.

Thus, our study indicates that a combination of flax seed oil, *Shorea robusta* resin and *Yashada bhasma* can be useful in wound contraction, improvement of tensile strength and augmentation in hydroxyproline content or collagen content. These properties together make this combination a potential candidate for anti-aging activities especially for better skin health.

## Figures and Tables

**Table 1:** Composition of five variants of anti-aging creams.

S. No	Cream variant code	Main ingredients
1	NATAF 001	*Shorea robusta* resin extract in flax seed oil
2	NATAF 002	*Shorea robusta* resin extract in flax seed oil + *Amalaki* fruit extract
3	NATAF 003	*Shorea robusta* resin extract in flax seed oil + cow ghee
4	NATAF 004	*Shorea robusta* resin extract in flax seed oil + *Yashada bhasma*
5	NATAF 005	*Shorea robusta* resin extract in flax seed oil + *Amalaki* fruit extract + cow ghee + *Yashada bhasma*

**Table 2:** Results of physicochemical analysis.

Test parameters	Cow ghee	Flax seed oil	*Amalaki* fruit extract	*Shorea Robusta* resin	*Yashada bhasma*
Description	Semi solid, granular in texture, light yellow in color with sweet characteristic odor	Liquid, golden yellow in color with characteristic odor	Free flowing powder, light greenish brown in color with faint characteristic odor	Dried resin pieces, irregularly cylindrical in shape, longitudinally shriveled, brownish to gray in color with faint balsamic odor	Fine powder of light yellow color, odorless, tasteless
Solubility	Soluble in chloroform, hexane, Dichloromethane (DCM), toluene, sparingly soluble in methanol, insoluble in water	Soluble in chloroform, hexane, DCM, toluene, sparingly soluble in methanol, insoluble in water	Very slightly soluble in chloroform, hexane, DCM, soluble in water, methanol	Soluble in chloroform, hexane, DCM, methanol, slightly soluble in water	Insoluble in chloroform, hexane, DCM, methanol, water, freely soluble in 50% HCl
pH	Not applicable	Not applicable	3.11	5.80	7.29
Wt/ml	0.905	0.926	Not applicable	Not applicable	Not applicable
RI	1.4590	1.477	Not applicable	Not applicable	Not applicable
Loss on drying at 105°C (% by wt)	Not applicable	Not applicable	7.46	1.25	0.049
Total ash content (% by wt)	Not applicable	Not applicable	4.77	0.049	99.95
Acid value	0.66	0.83	Not applicable	Not applicable	Not applicable
Iodine value	5.80	5.89	Not applicable	Not applicable	Not applicable
Peroxide value	1.49	9.1	Not applicable	Not applicable	Not applicable
Saponification value	230.66	207.30	Not applicable	Not applicable	Not applicable
Baudouin test	Negative	Not applicable	Not applicable	Not applicable	Not applicable
Sieve analysis, % retention on 150 *μ*% retention on 75 *μ*	Not applicable	Not applicable	Not applicable	Not applicable	0.40, 0.59
Bulk density (gm/ml) After first tap After 50 taps	Not applicable	Not applicable	Not applicable	Not applicable	1.38, 2.35
Percentage vitamin C	Not applicable	Not applicable	7.57	Not applicable	
Percentage zinc content	Not applicable	Not applicable	Not applicable	Not applicable	79.32
Heavy metals (Hg, Cd, As, Pb)	Not applicable	Not applicable	<0.5 ppm	<0.5 ppm	<0.5 ppm
Microbial load(cfu/gm) TVC TFC	Not applicable	Not applicable	<100, <10	<10, <10	Not applicable

**Table 3:** Percentage ALA fatty acid for accelerated stability studies in test preparations.

Variants	Initial	1 month		2 months		3 months		6 months	
		RT	40°C 75% RH	RT	40°C 75% RH	RT	40°C 75% RH	RT	40°C 75% RH
NATAF 001	1.75	1.70	1.69	1.75	1.73	1.71	1.74	1.73	1.71
NATAF 002	1.78	1.73	1.69	1.67	1.63	1.75	1.69	1.77	1.75
NATAF 003	2.73	2.70	2.67	2.72	2.69	2.70	2.65	2.72	2.70
NATAF 004	1.74	1.73	1.73	1.71	1.70	1.69	1.72	1.70	1.70
NATAF 005	2.24	2.18	2.20	2.24	2.19	2.23	2.25	2.23	2.20

**Table 4:** Effect of NATAF creams on wound contraction in excision wound model.

Group	Percentage wound contraction			
	Day 3	Day 5	Day 7	Day 9

01	15.62 ± 2.47	41.32 ± 2.94	74.34 ± 0.94	84.49 ± 0.67
02	12.68 ± 3.22	42.54 ± 1.53	74.15 ± 0.80	84.56 ± 0.40
03	11.78 ± 2.08	39.07 ± 2.76	73.61 ± 0.94	83.56 ± 0.32
04	11.29 ± 3.38	40.87 ± 3.05	75.22 ± 0.90	84.95 ± 0.35
05	13.71 ± 3.32	44.06 ± 2.08	81.00 ± 1.86*	91.71 ± 1.00*
06	13.81 ± 3.92	44.12 ± 2.19	81.04 ± 1.23*	91.51 ± 0.77*

Percentage wound contraction is calculated with respect to the wound area on Day 1 (at the time of induction) with respect to each animal. Values are mean ± SEM, *n* = 12. *P <  .01 versus control.
